# Structural Basis for the Oligomerization-State Switch from a Dimer to a Trimer of an Engineered Cortexillin-1 Coiled-Coil Variant

**DOI:** 10.1371/journal.pone.0063370

**Published:** 2013-05-14

**Authors:** Saša Bjelić, Mara Wieser, Daniel Frey, Christian U. Stirnimann, Mark R. Chance, Rolf Jaussi, Michel O. Steinmetz, Richard A. Kammerer

**Affiliations:** 1 Laboratory of Biomolecular Research, Paul Scherrer Institut, Villigen, Switzerland; 2 Center for Proteomics & Bioinformatics, Case Western Reserve University, Cleveland, Ohio, United States of America; 3 Swiss Light Source, Paul Scherrer Institut, Villigen, Switzerland; Indian Institute of Science, India

## Abstract

Coiled coils are well suited to drive subunit oligomerization and are widely used in applications ranging from basic research to medicine. The optimization of these applications requires a detailed understanding of the molecular determinants that control of coiled-coil formation. Although many of these determinants have been identified and characterized in great detail, a puzzling observation is that their presence does not necessarily correlate with the oligomerization state of a given coiled-coil structure. Thus, other determinants must play a key role. To address this issue, we recently investigated the unrelated coiled-coil domains from GCN4, ATF1 and cortexillin-1 as model systems. We found that well-known trimer-specific oligomerization-state determinants, such as the distribution of isoleucine residues at heptad-repeat core positions or the trimerization motif Arg-h-x-x-h-Glu (where h = hydrophobic amino acid; x = any amino acid), switch the peptide’s topology from a dimer to a trimer only when inserted into the trigger sequence, a site indispensable for coiled-coil formation. Because high-resolution structural information could not be obtained for the full-length, three-stranded cortexillin-1 coiled coil, we here report the detailed biophysical and structural characterization of a shorter variant spanning the trigger sequence using circular dichroism, anatytical ultracentrifugation and x-ray crystallography. We show that the peptide forms a stable α-helical trimer in solution. We further determined the crystal structure of an optimised variant at a resolution of 1.65 Å, revealing that the peptide folds into a parallel, three-stranded coiled coil. The two complemented R-IxxIE trimerization motifs and the additional hydrophobic core isoleucine residue adopt the conformations seen in other extensively characterized parallel, three-stranded coiled coils. These findings not only confirm the structural basis for the switch in oligomerization state from a dimer to a trimer observed for the full-length cortexillin-1 coiled-coil domain, but also provide further evidence for a general link between oligomerization-state specificity and trigger-sequence function.

## Introduction

As a result of its simple architecture consisting of just one type of secondary structure, the α-helical coiled coil is considered a paradigm for studies aimed at understanding the fundamental principles that govern protein stability, folding and oligomerization [1], [2]. Such studies have resulted in the extensive use of coiled coils for the rational design of multi-stranded structures in applications as diverse as basic research, biotechnology, nanotechnology, materials science and medicine [1]. For example, designed two- and three-stranded coiled coils were successfully used as lead molecules to target the adenomatous polyposis coli tumor-suppressor protein implicated in colorectal cancers and to inhibit HIV infection, respectively [3], [4]. However, the factors that control coiled-coil folding and oligomerization are not yet well understood. Amongst such factors, “trigger” sequences are known to play an important role in coiled-coil formation [5]–[10]. They comprise short, distinct amino-acid sequences, many of which fold into moderately stable monomeric α-helices prior to the formation of coiled-coil structures. Apparently, the function of a trigger sequence is to present key residues in a coiled coil-like conformation, which serves as a scaffold for the recognition and in-register alignment of partner helices. Interacting helices then “zip up” along the molecule to form a stable coiled-coil structure.

Several determinants that control the oligomerization state of coiled coils have been identified and studied in great detail. Major roles are played by the specific placement of hydrophobic core residues, in particular isoleucine and leucine, and their distribution generally correlates well with the oligomerization state of coiled coils [11]–[13]. An accumulation of isoleucine and leucine residues at the heptad repeat **a** and **d** core positions, respectively, favors the formation of dimers whereas the reverse arrangement results in tetrameric structures. In contrast, a more even distribution of isoleucine at both the **a** and **d** positions facilitates trimer formation.

Interhelical interactions between side chains of residues at the **e** and **g** positions as well as the packing of these amino acids against the hydrophobic **a** and **d** core residues also contribute significantly to oligomerization-state specificity of coiled coils [1], [2]. This is exemplified by the trimerization motif Arg(**g**)-h(**a**)-x-x-h(**d**)-Glu(**e**) (denoted R-hxxhE where h(**a**) = Ile, Leu, Val, Met; h(**d**) = Leu, Ile, Val; x = any amino acid residue) that specifies a three-stranded, parallel topology of coiled-coil domains [14]–[16]. The trimerization driving force of the motif can be explained by optimal side chain–side chain interactions whereby the strictly conserved arginine and glutamate residues form a distinct, bifurcated, interhelical salt-bridge network and participate in the formation of the hydrophobic core by establishing tight packing interactions to the neighbouring residues at the **a** and **d** positions through their aliphatic moieties.

**Figure 1:**
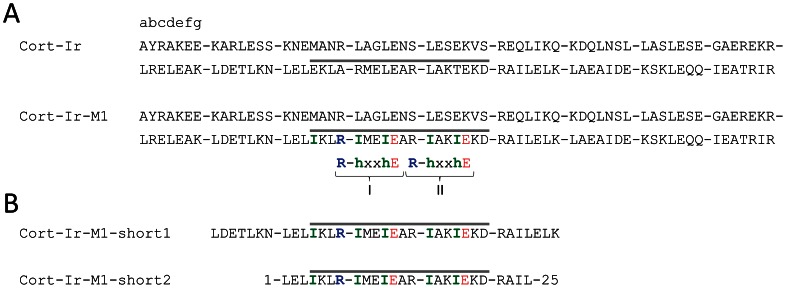
Design of Cort-Ir-M1 variants. (A) Amino-acid sequence of the wild-type Cort-Ir coiled-coil dimer and its trimeric mutant Cort-Ir-M1. Heptad repeats (abcdefg) are indicated and shown as blocks. The experimentally determined trigger sequence is highlighted by a gray bar. Arginine and glutamate residues of the two complemented trimerization motifs I and II and additional trimer-specific isoleucine substitutions are highlighted in colour according to the amino acids’ physicochemical properties: blue, positively charged; red, negatively charged; green, hydrophobic, h, hydrophobic, x, any amino acid. (B) Amino- acid sequence of Cort-Ir-M1-short1 and Cort-Ir-M1-short2.

An open issue is that the presence of a specific oligomerization-state determinant frequently does not correlate with the corresponding coiled-coil topology. Although the trimerization motif R-hxxhE is predominantly found in protein families harboring parallel, three-stranded coiled-coil domains, it is also present in some dimers and antiparallel trimers [15]. We have recently addressed this issue and identified a general link between coiled-coil oligomerization-state specificity and trigger-sequence function [14]. By using the archetype coiled-coil domain of the yeast transcriptional activator GCN4 as a model system, we showed that trimer-specific oligomerization-state determinants such as the trimerization motif or isoleucine residues at the heptad-repeat **a** and **d** positions switch the peptide’s topology from a dimer to a trimer only when inserted into the trigger sequence. We successfully confirmed our results in two other, unrelated coiled-coil dimers, ATF1 and cortexillin-1. Because of its substantial size of 18 continuous heptad repeats, we used a combination of trimer-specific determinants to switch the cortexillin-1 coiled coil from a dimer to a trimer. Accordingly, we rationally substituted residues to complement two ideal trimerizer motifs of the type R-IxxIE and introduced one additional hydrophobic core isoleucine residue in the trigger sequence of the cortexillin-1 coiled coil (referred to as Cort-Ir-M1; Figure 1). These substitutions were designed to span the entire trigger sequence [14].

Because high-resolution structural information could not be obtained for the full-length Cort-Ir-M1 trimer, the aim of the present study was to perform a detailed structural characterization of two fragments spanning the trigger sequence of the trimeric Cort-Ir-M1 coiled coil using circular dichroism, anatytical ultracentrifugation and x-ray crystallography. We provide the structural basis for the observed switch in oligomerization state of the cortexillin-1 coiled coil from a dimer to a trimer, and therefore provide further evidence for the general link between coiled-coil oligomerization-state specificity and trigger-sequence function.

## Materials and Methods

### Cloning and Protein Preparation

The GFP-Cort-Ir-M1-short1 and Cort-Ir-M1-short2 protein (Figure 1) were expressed from our in-house constructed expression vectors [17] using the T7 promoter. The N termini of Cort-Ir-short1 and Cort-Ir-short2 were fused to 6xHis-tagged GFP with a prescisson recognition site and 6xHis-tagged *E. coli* thioredoxin A containing a thrombin cleavage site, respectively. To facilitate AUC measurements of the untagged protein, one tryptophan residue was introduced at C terminus of the Cort-Ir-M1-short1 variant. The constructs were verified by DNA sequencing (GATC, Germany). GFP-Cort-Ir-short1 and Cort-Ir-short2 were produced in *E. coli* BL21(DE3) and T7 Express Crystal cells (New England Biolabs), respectively, using LB medium. After induction with 1 mM IPTG at an OD_600_ of 0.7, cultures were incubated overnight at 20°C. The proteins were purified by Ni-NTA-chromatography and size exclusion chromatography. Cort-Ir-M1-short1 and Cort-Ir-M1-short2 were separated from their respective fusion proteins by thrombin and prescission protease (GE Healthcare) cleavage followed by size exclusion chromatography in PBS (2.7 mM KCl, 1.5 mM KH_2_PO_4,_ 136.9 mM NaCl, 8.9 mM Na_2_HPO_4_) and 30 mM Tris-HCl pH 7.5, 300 mM NaCl, 6 mM imidazole, 5 mM β-mercaptoethanol, respectively. For crystallization trials, Cort-Ir-M1-short2 was concentrated to 3 mg/ml.

### Circular Dichroism (CD) Spectroscopy

CD experiments were performed on a Chirascan-Plus instrument (Applied Photophysics Ltd.) equipped with a computer-controlled Peltier element using a cuvette of 10-mm optical path length. Thermal unfolding profiles of Cort-Ir-M1-short1 were recorded at a concentration of 13 µM (monomer equivalents) in PBS, by continuous heating at 1°C min^−1^.

### Analytical Ultracentrifugation (AUC)

Absorption AUC sedimentation equilibrium experiments were performed in a Beckman-Coulter Optima XL-1 (Beckman-Coulter, Brea, CA, USA) at 235 nm/25 krpm and 411 nm/18 krpm for Cort-Ir-M1-short1 and GFP-Cort-Ir-M1-short1, respectively. All experiments were performed at 20°C in PBS. Data were analyzed using the UltraScannII software suite [18].

Fluorescence-monitored AUC sedimentation velocity experiments were performed in a Beckman-Coulter Optima XL-1 ultracentrifuge (Beckman-Coulter, Brea, CA, USA) equipped with an Aviv fluorescence detection system [19]. GFP-Cort-Ir-M1-short1 samples were diluted into PBS buffer supplemented with 0.2 mg/ml lysozyme to prevent adhesion of protein onto the AUC cell surface. The experiments were performed at 45 krpm and 20°C, and the sedimentation coefficients were determined using the SedFit software package [20].

The dissociation constant of GFP-Cort-Ir-M1-short1 was calculated using a two-state-model, assuming only monomeric and trimeric species as there was no experimental evidence for the presence of significant amounts of dimers.

### Crystal Structure Determination

Crystals of Cort-Ir-M1-short2 were grown at 20°C by using the sitting drop method. Initial hits were obtained using commercially available screens (Hampton Research). Several rounds of crystal optimization were performed at the crystallization facility of the Swiss Light Source (Villigen PSI). Best crystals were obtained by mixing equal volumes of protein (1.3 mM) with the reservoir solution containing 0.3 M potassium hydrogen phosphate, 20% glycerol and 16% PEG 8000. The crystals were directly frozen at the beamline X06SA of the Swiss light Source in a 100 K nitrogen stream. Data collection was performed according to the fine φ-slicing strategy [21].

The structure of Cort-Ir-M1-short2 was solved by molecular replacement employing the Phaser software package implemented in the PHENIX software suite [22], [23] and using the matrilin-1 structure as a search model (PDB ID 1AQ5). To this end, the data were cut at I/σ = 2 and CC_half_ = 1.

The structure refinement was performed using PHENIX and TLS Motion Determination [24]. Data collection and refinement statistics are given in Table 1. Figures were prepared with PyMOL [25]. Coordinates of the Cort-Ir-M1-short2 have been deposited in the Protein Data Bank with accession codes 4J4A.

**Table 1:** Data collection and refinement statistics.

	Cort-Ir-M1-short2
Data collection^a^	Native
Space group	P2_1_2_1_2_1_
Unit cell (Å, °)	a = 41.88 b = 78.05 c = 114.99 α = β = γ = 90°
Wavelength (Å)	1
Resolution (Å)	46.29−1.60
	(1.65−1.60)
R_meas_ (%)	8.6 (83.8)
Completeness^a^ (%)	99.9 (99.8)
Redundancy a	26.2
I/σ(I)^a^	31 (1.9)
CC_half_ b	1.00 (0.91)
**Refinement statistics**	
Resolution (Å)	46.29−1.65 (1.70−1.65)
No. unique reflections	46230 (4640)
R_ work_/R_ free_ (%)	21.3/25.8
**Average B factor (Å^2^)**	
Protein atoms	34.9
Water	34.2
**RMSD from ideal values**	
Bonds/angles (Å/°)	0.015/1.542
**Ramachandran plot statistics** c	
Favored regions (%)	98.9
Allowed regions (%)	0.7
Disallowed (%)	0.4

a Values in parentheses correspond to the highest resolution shell.

b As defined by Karplus & Diederichs [34].

c Statistics from Molprobity [35].

## Results

### Cort-Ir-M1-short1 Forms a Stable α-helical Trimer in Solution

To confirm on the molecular level that the observed switch of Cort-Ir-M1 from a dimer to a trimer was the result of the introduction of functional trimer-specific oligomerization-state determinants, we aimed to elucidate the crystal structure of the full-length coiled-coil domain. However, extensive crystallization trials using Cort-Ir-M1 resulted in crystals that diffracted to low resolution only and were not suitable for the collection of a full dataset. We therefore produced two shorter fragments of the Cort-Ir-M1 mutant, denoted Cort-Ir-M1-short1 and Cort-Ir-M1-short2, which span the entire trigger sequence and contain all the amino-acid substitutions that were introduced to switch the full-length coiled-coil domain from a dimer to a trimer (Figure 1; [14]). Prior to initiating crystallization trials, we characterized Cort-Ir-M1-short1 in detail by biophysical methods.

**Figure 2:**
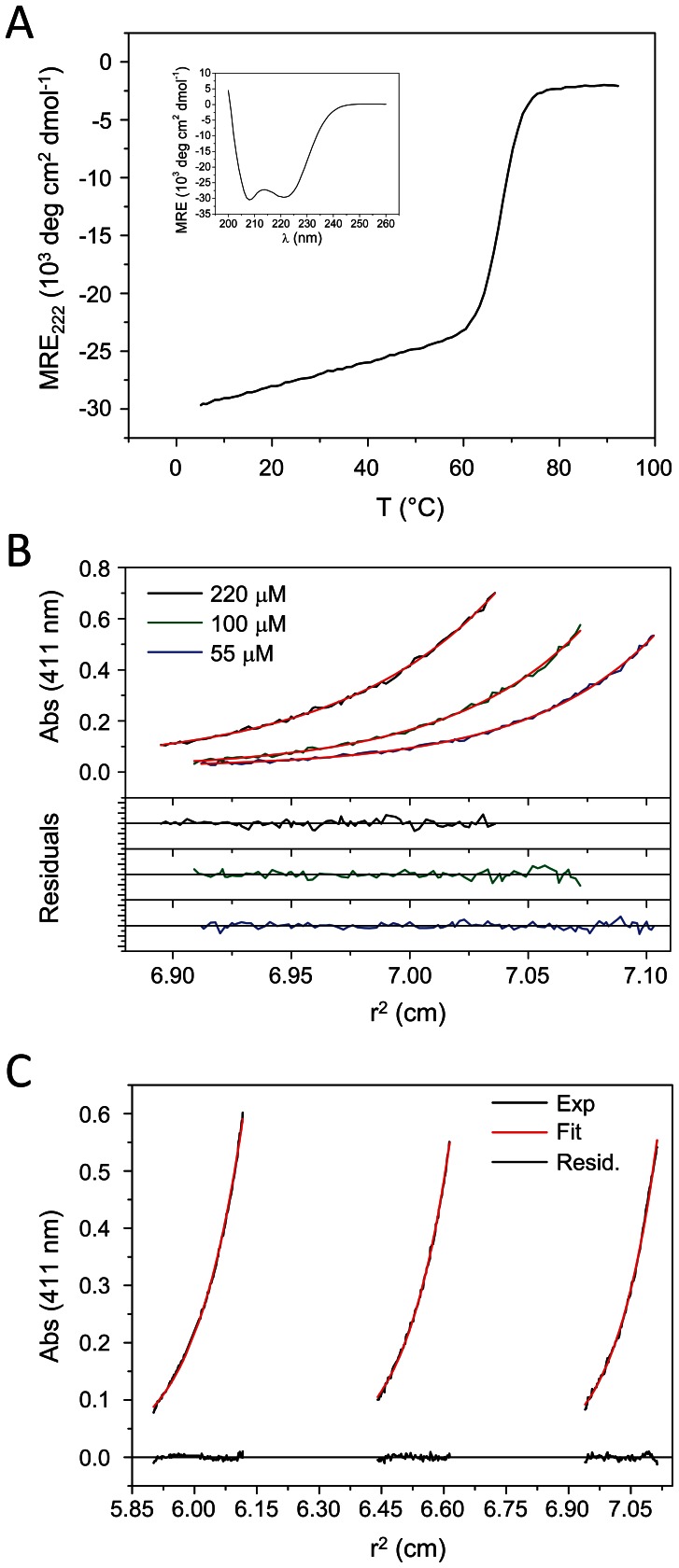
CD and sedimentation equilibrium analysis of Cort-Ir-M1-short1 and GFP-Cort-Ir-M1-short1. (A) Thermal unfolding profile of Cort-Ir-M1-short1 monitored by CD. The CD signal at 222 nm was followed by increasing the temperature at a rate of 1°C/min. The midpoint of unfolding, T_m_, is centred at 69°C at a protein concentration of 13 µM. Inset, CD spectrum of Cort-Ir-M1-short1 at 5°C. (B) Analysis of the oligomerization state of GFP-Cort-Ir-M1-short1 by sedimentation-equilibrium analysis. The protein was analyzed at concentrations of 55, 110 and 220 µM in PBS. (C) Analysis of the oligomerization state of Cort-Ir-M1-short by sedimentation-equilibrium analysis. The protein was analyzed at a concentration of 5 µM in PBS. The data were collected at 235 nm. All AUC data were globally fitted according to a single ideal species model and in all cases the derived masses are consistent with the presence of trimmers only. Concentrations refer to monomer equivalents.

CD spectroscopy was used to test for the secondary structure of the Cort-Ir-M1-short1 peptide. The far-ultraviolet CD spectrum recorded from Cort-Ir-M1-short1 indicated a substantial amount of α-helicity at a concentration of 13 µM (monomer equivalents) and at a temperature of 5°C (Figure 2A). The CD spectrum was characteristic of a coiled coil as judged from the similarly pronounced minima at 208 and 222 nm (Figure 2A). A [θ]_222_ value of approximately −30′000 deg cm^2^ dmol^−1^ indicated a degree of α-helicity of ∼90% [26]. The stability of the peptide was assessed by a thermal unfolding profile recorded by CD at 222 nm (Figure 2A, inset). The thermal unfolding profile exhibited the sigmoid shape typical of coiled coils, implying a two-state transition [27]. At a peptide concentration of 13 µM (monomer equivalents), the monophasic profile showed a T_m_ centered at 69°C (Figure 2A), demonstrating considerable stability.

**Figure 3:**
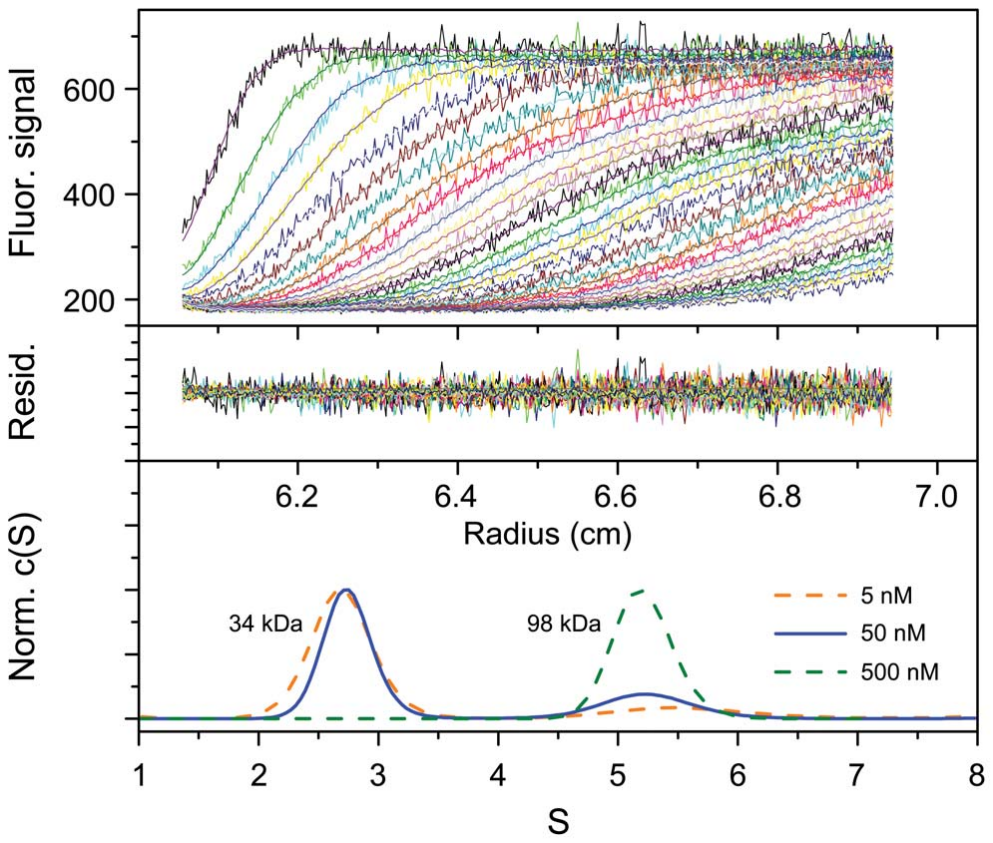
Sedimentation velocity analysis of GFP-Cort-Ir-M1-short1. Fluorescence-monitored AUC sedimentation velocity of GFP-Cort-Ir-M1-short1. The protein concentrations were 5, 50 and 500 nM (monomer equivalents). The fluorescence of GFP was monitored at 488 nm. Traces and residuals are shown for the experiment carried out at a protein concentration of 50 nM. The distribution of sedimentation coefficients indicates the presence of only monomeric and trimeric species only. The fitted masses for single ideal species are listed in the lower panel. The dissociation constant, K_D_, of GFP-Cort-Ir-M1-short was estimated to be 10^−14^ M^−2^.

To determine the oligomerization state of Cort-Ir-M1-short1 in solution, different concentrations of the peptide were analyzed by AUC (Figure 2, B and C). Sedimentation equilibrium yielded average molecular masses of 13.2 kDa, which is consistent with the formation of a homotrimer (calculated monomer molecular mass is 4.5 kDa). Next we determined the dissociation constant, K_D_, of Cort-Ir-M1-short1 by fluorescence sedimentation velocity experiments. To this end, we produced an N-terminal GFP-fusion version of the coiled-coil domain (denoted GFP-Cort-Ir-M1-short1). The sedimentation profile of GFP-Cort-Ir-M1-short1 at 500 nM monomer equivalents revealed only a single peak centred at ∼5.2 S, which we assigned to a GFP-Cort-Ir-M1-short1 trimer (sequence derived molecular mass of 99 kDa for the trimer; molecular mass of 98 kDa derived from S value) (Figure 3). At 5 nM concentration, the lowest concentration that was measurable in a reproducible manner, only one peak centred at 2.7 S was observed, corresponding to 33 kDa. At 50 nM concentration, we observed two peaks centred at ∼2.8 and ∼5.2 S. The second peak corresponded to the already observed one for the GFP-Cort-Ir-M1-short1 trimer; the first peak was assigned to a GFP-Cort-Ir-M1-short1 monomer (calculated molecular mass of 33 kDa; molecular mass of 34 kDa derived from S value). We did not find any indication of dimeric GFP-Cort-Ir-M1-short1 species in this experiment. We estimated the K_D_ of GFP-Cort-Ir-M1-short1 (3 monomers ↔ 1 trimer) to be 10^−14^ M^−2^. Collectively, these data demonstrate that Cort-Ir-M1-short1 forms a stable three-stranded coiled coil in solution.

### Structural Basis for Trimer Formation of Cort-Ir-M1-short

**Figure 4:**
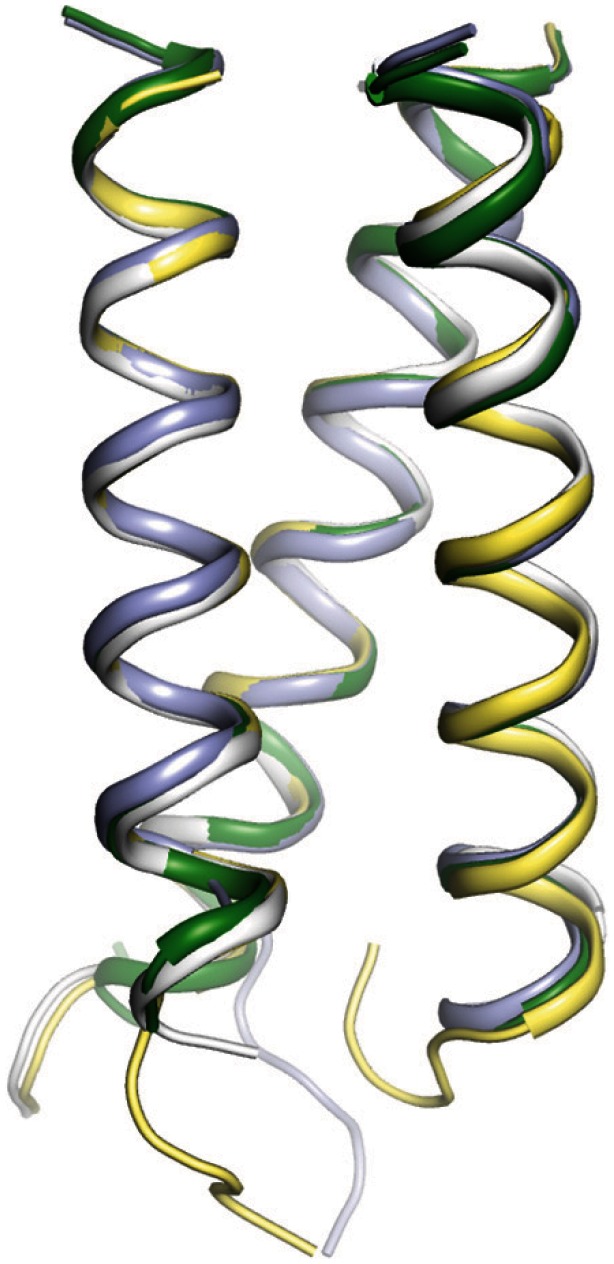
X-ray crystal structure of the Cort-Ir-M1-short2 coiled-coil trimer. Side view with the N terminus on top. The four Cort-Ir-M1-short2 copies of the asymmetric unit of the crystal have been superimposed in different colours.

We obtained well-diffracting crystals of Cort-Ir-M1-short2 and determined its high-resolution crystal structure to a resolution of 1.65 Å (Table 1). As expected, the peptide forms a trimer with characteristics typical of left-handed parallel coiled coils (Figure 4). The asymmetric unit of the crystal contained four copies of the Cort-Ir-M1-short2 trimer; analysis of these four copies revealed differences between them ranging from 0.45–0.65 Å RMSD over >52 Cα-atoms.

**Figure 5:**
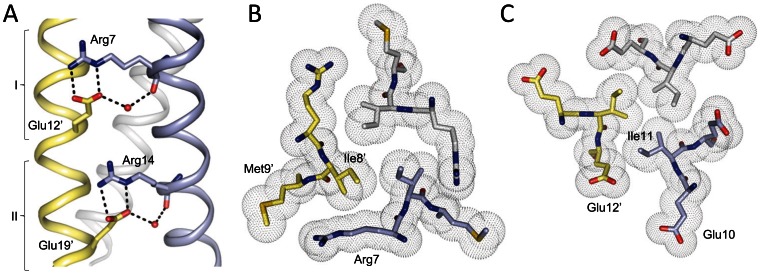
Prominent structural features seen in the Cort-Ir-M1-short2 coiled-coil trimer. (A) Side view of the salt-bridge networks formed between Arg7/14 and Glu12′/19′, and water-mediated hydrogen bond between Glu12′/19′:Oε2 and Arg7/14:O. The position of the two trimerization motifs I and II is indicated. (B) End-on view of the **a**2 layer showing the shielding of the Ile8 residues from solvent by the aliphatic side-chain moieties of Arg7. (C) End-on view of the **d**2 layer showing the hydrophobic packing between Ile11 and the aliphatic side-chain moieties of the Glu12 residues. Side chains of residues are shown in sticks representation and van der Waals spheres (B and D), the water molecules as small red spheres (A), and monomers A, B and C are shown as Cα-traces. Oxygen and nitrogen atoms are coloured in red and blue, and carbon atoms in cyan, yellow and grey for monomers A, B and C, respectively. The amino-acid sequence and sequence features of Cort-Ir-M1-short2 is shown in Figure 1.

The most prominent feature seen in the crystal structure of Cort-Ir-M1-short2 is a network of surface salt bridges and internal hydrophobic packing interactions formed by residues comprising the two trimerization motifs of the type R-IxxIE (Figure 5A). These interactions are virtually identical to those observed for the parallel, three-stranded coiled-coil domains of coronin-1 [15] and ccβ [28], or the trimeric GCN4 mutant GCN4p-M3 [14]. Accordingly, the conformations of side chains and the position of the water molecule between Arg7 and Glu12, and Arg14 and Glu19 can be superimposed almost perfectly onto the trimerization motifs of the coronin-1 coiled coil, ccβ and GCN4p-M3, demonstrating structural conservation of the motif (not shown). The extension of the characteristic bifurcated contact between Arg14N*ε*, Nη2 at position **g** of one chain and Glu19′O*ε*1, Oε2 at position **e**′ of the neighboring chain by an additional interaction between Arg14N*η*1 and Glu12’O*ε*1 at position **e**′ that is seen in GCN4p-M3 is not observed in the Cort-Ir-M1-short2 structure because Glu12’ is part of the first trimerization motif (Figure 5A). Tight interhelical hydrophobic contacts involve the side chain of Ile8′ at position **a**′ of the first trimerization motif, which is optimally shielded from solvent exposure by the aliphatic side-chain portion of Arg7 (Figure 5B). Similarly, Ile11 at position **d** of the second trimerization motif packs against the hydrophobic side chain moiety of Glu12′ at position **e**′ (Figure 5C). Notably, a similar optimal packing of the hydrophobic core and the presence of an extensive network of surface salt bridges are typically observed in thermostable proteins [29].

The interior packing of Cort-Ir-M1-short2 at layers **a**2,3 and **d**1,2,3 also provides a structural basis for the switch of the oligomerization state from a dimer to a trimer. In the structure, the five introduced isoleucine residues can be accommodated in the most preferred rotamer at the hydrophobic core positions. Accordingly, the acute packing geometries of the five isoleucine residues are very similar to those seen in the crystal structure of the trimeric GCN4-pII variant [12], in which all **a** and **d** positions are occupied by isoleucine residues (not shown).

Taken together, the two complemented R-IxxIE trimerization motifs and the additional hydrophobic-core isoleucine residue in Cort-Ir-M1-short2 adopt the conformations seen in other extensively characterized, parallel, three-stranded coiled coils, such as coronin-1, ccβ or GCN4p-M3. They therefore not only confirm the structural basis for the switch in oligomerization state from a dimer to a trimer observed for the full-length Cort-Ir-M1 coiled-coil domain, but also provide further evidence for a general link between oligomerization-state specificity and trigger sequence function.

## Discussion

With the overall aim to understand the specificity of coiled-coil formation at the molecular level, we here provide a structural explanation for the observed switch in oligomerization state of the cortexillin-1 coiled coil from a dimer to a trimer and further generalize the relationship between coiled-coil oligomerization-state specificity and trigger-sequence function. Our study therefore represents a confirmation and extension of existing sequence-to-structure rules [1], [2]. Such detailed information on coiled-coil sequence-to-structure rules should be beneficial for retrospective interpretation of existing results and could form a basis for design of new molecular entities.

A well-documented example is the application of a coiled coil-based strategy for the investigation of the activation mechanisms of prokaryotic and eukaryotic transmembrane receptors. These studies have shown that dimerization is not always sufficient to induce autophosphorylation and that additional conformational changes such as, for example, rotation of the kinase domains relative to each other are required for activation. A coiled-coil based approach has been used to test the dimerization-rotation model for several homodimeric proteins (for a representative example, see [30]). Typically, the extracellular domain of a given receptor is replaced by the short dimeric coiled coil of the *S. cerevisiae* transcription factor Put3 and fused to its transmembrane domain and intracellular domain. By removing residues of the transmembrane domain at the junction to the coiled coil, designed variants with seven different rotational conformations of the cytosolic domains relative to each other were generated by imposing the Put3 heptad-repeat pattern [30]. However, in some cases the Put3 coiled coil might not be stable or specific enough to impose the conformations on the transmembrane domain and/or the cytoplasmic domain as it possesses a rather moderate stability of only 40°C at a monomer concentration of 100 µM (R.A. Kammerer, unpublished result). This finding suggests that results obtained with Put3 have to be interpreted with caution. However, on the basis of our extended sequence-to-structure rules it should be possible to optimize Put3 for studies of transmembrane receptors.

Angiopoietins are the ligands of Tie2 and have potential therapeutic applications in angiogenesis and prevention of vascular leakage. However, the large-scale production of recombinant angiopoietins, in particular angiopoietin-1, is hindered by the aggregation and insolubility of the proteins [31]. We and others have produced soluble angiopoietin-1 variants by replacing the native oligomerization domains by coiled-coil domains from other proteins [31]. Our designed trimeric variant using the coiled-coil domain of matrilin-1 as a fusion partner formed a mixture of trimers and tetramers and phosphorylated Tie2 to the same extent as the native protein. However, the minimal oligomerization state of angiopoietin-1 required for Tie2 activation is currently unknown. The Cort-Ir-M1-short sequence appears to be an excellent candidate fusion partner to address this question.

Integrins comprise a large family of heterodimeric adhesion receptors consisting of α and β subunits. They link the extracellular matrix to the cytoskeleton, a process in which the structure of cytoplasmic domains of integrin β subunits plays a key role [32]. The majority of integrin ligands result in receptor clustering and it has been suggested that isolated cytoplasmic β subunit domains resemble ligand-occupied integrins. To mimic this process, Pfaff and colleagues fused cytoplasmic β subunit domains to a four heptad-repeat long particular coiled coil, K-LEALEGR-LDALEGK-LEALEGK-LDALEG, with the aim to generate parallel homodimers [33]. Despite the authors found differences in the binding of clustered β subunit domains with regard to cytoskeletal proteins, their choice of the coiled coil appears not ideal for the design of specific, parallel dimers. The coiled coil contains leucine residues at both the **a** and **d** positions, a distribution that was shown to poorly specify the oligomerization state and result in the formation of mixed oligomers, as well as parallel and antiparallel coiled-coil structures [11]. It should also be noted that the coiled coil contains a trimerization motif of the type R-LDALE, which is a strong determinant of specific trimer formation in such short coiled coils [14]–[16].

These examples highlight that the design of chimeric fusion proteins using coiled coils remains a challenge. They also emphasize the need for identifying further sequence-to-structure rules like the ones described in the present study. Such experimental findings should significantly improve the specificity of rationally designed coiled coils and consequently many applications using these popular oligomerization domains.
